# Comparison of oncological and perioperative outcomes of open, laparoscopic, and robotic nephroureterectomy approaches in patients with non-metastatic upper-tract urothelial carcinoma

**DOI:** 10.1371/journal.pone.0210401

**Published:** 2019-01-08

**Authors:** Hakmin Lee, Hak Ju Kim, Sang Eun Lee, Sung Kyu Hong, Seok-Soo Byun

**Affiliations:** 1 Department of Urology, Seoul National University Bundang Hospital, Seongnam, Korea; 2 Department of Urology, Seoul National University College of Medicine, Seoul, Korea; University of Washington, UNITED STATES

## Abstract

**Background:**

To compare the oncological and perioperative outcomes of different nephroureterectomy approaches in patients with non-metastatic upper tract urothelial carcinoma (UTUC).

**Methods:**

We retrospectively analyzed the data of 422 patients who underwent open, laparoscopic, or robotic nephroureterectomy for non-metastatic UTUC. Perioperative and postoperative survival outcomes were compared using Kaplan-Meier analyses and Cox-proportional hazard models.

**Results:**

Of the patients, 161, 137, and 124 were treated with an open, laparoscopic, and robotic approach, respectively. Laparoscopic and robotic approaches involved significantly less blood loss (p = 0.001), shorter hospital stay (p < 0.001), and longer operation time (p < 0.001) compared with the open approach. There were no significant differences in intraoperative complications (open, 8.1%; laparoscopic, 5.1%; robotic, 7.3%; p = 0.363) or early postoperative complications (open, 14.9%; laparoscopic, 14.6%; robotic, 13.7%; p = 0.880). The laparoscopic and robotic groups showed significantly less postoperative analgesic use (p = 0.015). The robotic group showed significantly longer progression-free, cancer-specific, and overall survivals than the open approach group on univariate Kaplan-Meier analysis, but surgery type was not significantly associated with survival outcomes per multivariate Cox proportional tests (all p-values > 0.05).

**Conclusion:**

The laparoscopic and robotic approaches yielded better perioperative outcomes, such as less intraoperative bleeding, shorter hospital stays, less analgesic usage, and non-inferior oncological outcomes, compared with the open approach. Further prospective studies are needed to compare these surgical techniques.

## Introduction

Upper tract urothelial cancer (UTUC) is a relatively uncommon malignancy compared with bladder cancer, which accounts for approximately 5% of all urothelial cell malignancies [1]. A relatively high incidence of UTUC has been reported in some Asian countries compared with some Western countries [2–3]. The Taiwan Cancer Registry Annual Report in 2013 reported the incidence of UTUC per 100,000 persons as 4.09 in men and 4.37 in women [4]. Although bladder cancer and UTUC have common histological backgrounds, UTUC shows more aggressive clinical behaviors and has an estimated 5-year overall mortality rate of 23% [5]. Moreover, the prognosis of muscle-invading UTUC is more catastrophic, showing a 5-year cancer-specific survival of <50% for T2/T3 disease and <10% for T4 disease [6–7].

Radical nephroureterectomy with bladder cuff excision is the gold standard treatment for non-metastatic UTUC [5]. During the past couple of decades, minimally invasive surgeries such as laparoscopic and robot-assisted approaches have been widely accepted for treating UTUC. Minimally invasive surgeries are known to have certain advantages including better cosmetic results from small skin incision and less bleeding from the pneumoperitoneum during surgery. Given that the robotic and laparoscopic approaches are commonly performed inside the peritoneal space, there has been concern about the possible risk of intraperitoneal rupture or tumor spillover during such surgeries, particularly in patients with large tumors. However, previous studies showed comparable oncological outcomes and additional advantages of those minimally invasive techniques including less postoperative pain, less intraoperative blood loss, and a shorter recovery time [8–9]. However, most studies were limited by their small number of subjects and such studies did not compare all available surgical approaches. Therefore, we tried to compare the actual oncological and perioperative outcomes of the three surgical approaches (i.e., open, laparoscopic, robotic) in patients with non-metastatic UTUC.

## Material and methods

After approval (B-1805/466-116) for the study was obtained from the ethical review board of Seoul National Bundang Hospital, we retrospectively analyzed the medical records of 459 patients who underwent surgery for UTUC between September 2004 and June 2017 in a single tertiary center in South Korea. All data were anonymized before accession and thus the requirement for informed consent was waived by the ethical committee, considering the retrospective nature of this study. After excluding 37 patients with other malignancies (n = 19) or metastatic diseases (n = 15) and those with incomplete information (n = 4), we finally included 422 subjects. Clinical and pathological information was acquired from a prospectively maintained database of our institution. Lymph node dissection was performed in cases of enlarged lymph nodes according to preoperative images or intraoperative findings suggestive of lymph node invasion. Pathologic stages and cellular grades were defined according to the 2002 American Joint Committee of Cancer TNM classification and the World Health Organization International Society of Urological Pathology consensus classification, respectively [10–11]. The surgical approach was selected on the basis of each surgeon’s clinical decision, with consent obtained from each patient. The open approach was performed via two different incisions: a flank incision for nephrectomy and an additional abdominal incision for bladder cuff excision. The laparoscopic approach was performed through transperitoneal access using one camera port over the lateral rectus margin at the umbilicus level and two additional working ports. As in the open approach, bladder cuff excision was also performed through an additional abdominal incision using the laparoscopic approach. The robotic approach was performed in a pure robotic fashion. If there was tumor involvement in the distal ureter or ureterovesical junction, we also excised the bladder cuff through an additional abdominal excision.

Postoperative follow-up was performed every 3–4 months during the first 2 years, every 6 months until the fifth year, and annually thereafter. Disease progression was identified when there was radiologic or pathologic evidence of local recurrence, distant metastasis, or mortality from UTUC. Mortality data were retrieved from medical record review and from the database of the Korean National Statistical Office. Progression-free, cancer-specific, and overall survival rates were calculated from the date of surgery to the date of progression, cancer-specific mortality, and all-cause mortality. The salvage chemotherapy was defined when the chemotherapy was given six months after the surgery.

Clinical and pathological characteristics were compared between the groups using analysis of variance and the chi-square test. Univariate and multivariate logistic regression tests were performed to analyze the associations between variables. Kaplan-Meier analyses with log-rank tests were used to compare survival outcomes between the groups, whereas multivariate Cox proportional hazard analyses were used to reveal the predictors of postoperative survival outcomes using the following co-variates: age, sex, body mass index, ECOG score, T stage, tumor size, cellular grade, Charlson’s comorbidity index, multifocality of the tumor, lymphadenectomy, and lymph node invasion. SPSS version 19.0 (SPSS, Chicago, IL, USA) was used for statistical analyses; p values were two-sided, and p-values <0.05 were considered statistically significant.

## Results

The clinical characteristics of the entire patient cohort are summarized in Table 1. There were 161 patients treated by the open approach, 137 by the laparoscopic approach, and 124 by the robotic approach. Between 2004 and 2009, most surgeries were performed by the open approach, but the number of robotic surgeries exceeded the number of open and laparoscopic surgeries between 2015 and 2018, with a significant between-group difference (p < 0.001). The median age was 69 years (interquartile range [IQR], 62–75), median tumor size was 3.5 (IQR, 2.4–5.3), and median follow-up time was 24 months (IQR, 10–59) in the entire cohort. There were no significant intergroup differences in preoperative characteristics including age, body mass index, history of diabetes, or performance score (all p-values > 0.05). However, the open approach group showed significantly worse pathological characteristics in terms of large tumor size (p = 0.018), a high rate of multi-focal tumors (p = 0.013), higher pathologic stage (p = 0.001), more lymph node invasion (p < 0.001), and lymphovascular invasion (p < 0.001) (Table 2). Furthermore, there was significant difference in the follow-up period between the different treatment groups (p < 0.001).

**Table 1:** Clinical characteristics according to the type of surgery.

	Mean ± SD or Counts (percent of total)			
	Open	Laparoscopic	Robotic	p value
Number of subjects	161	137	124	
Age (years)	67.5 ± 10.2	68.6 ± 10.4	67.6 ± 11.3	0.642
BMI (kg/m^2^)	23.7 ± 2.8	23.9 ± 3.6	24.6 ± 2.9	0.062
Sex (male)	117 (72.7%)	97 (70.8%)	85 (68.5)	0.750
Diabetic mellitus	30 (18.6%)	26 (19.0%)	24 (19.4%)	0.988
Hypertension	83 (51.6%)	75 (54.7%)	57 (46.0%)	0.360
ECOG score > 1	5 (3.1%)	3 (2.2%)	1 (0.8%)	0.411
Tumor size (cm)	4.9 ± 4.2	4.4 ± 3.6	3.7 ± 2.0	0.018
Laterality (right)	85 (52.8%)	77 (56.2%)	67 (54.0%)	0.839
Preoperative hydronephrosis	115 (71.9%)	90 (65.7%)	83 (68.0%)	0.577
History of gross hematuria	107 (66.5%)	100 (73.0%)	86 (69.4%)	0.419
Years treated				< 0.001
2004–2009	80 (49.7%)	36 (26.3%)	3 (2.4%)	
2010–2015	70 (43.5%)	59 (43.1%)	25 (20.2%)	
2015—present	11 (6.8%)	42 (30.7%)	96 (77.4%)	

SD = standard deviation, BMI = body mass index; CIS = carcinoma in situ; ECOG = Eastern Cooperative Oncology Group

**Table 2:** Pathologic outcomes according to the type of surgery.

	Mean ± SD or Counts (percent of total)			
	Open	Laparoscopic	Robotic	p value
Number of subjects	161	137	124	
Tumor location				0.013
Intra-renal	84 (52.2%)	78 (56.9%)	63 (50.8%)	
Ureter	54 (33.5%)	53 (38.7%)	53 (42.7%)	
Both	23 (14.3%)	6 (4.4%)	8 (6.5%)	
Pathologic T stage				0.001
Ta	3 (1.9%)	2 (1.5%)	0 (0%)	
T1	34 (21.1%)	46 (33.6%)	41 (33.1%)	
T2	44 (27.3%)	40 (29.2%)	47 (37.9%)	
T3	63 (39.1%)	46 (33.6%)	32 (25.8%)	
T4	17 (10.6%)	2 (1.5%)	3 (2.4%)	
CIS only	0 (0%)	1 (0.7%)	1 (0.8%)	
Cellular grade				0.177
Grade I	1 (0.6%)	1 (0.7%)	0 (0%)	
Grade II	68 (42.2%)	74 (54.0%)	67 (54.0%)	
Grade III	92 (57.1%)	62 (45.3%)	57 (46.0%)	
Lymph node invasion	59 (36.6%)	27 (19.7%)	9 (7.3%)	< 0.001
Lymphovascular invasion	67 (41.6%)	35 (25.5%)	26 (21.0%)	< 0.001
Lymph node collected	10.0 ± 2.5	7.3 ± 4.9	6.4 ± 3.9	0.452
Number of positive nodes	1.1 ± 1.0	0.6 ± 0.8	1.2 ± 1.6	0.543
Follow-up periods	41.7 ± 3.3	38.1 ± 3.3	23.7 ± 2.1	< 0.001

SD = standard deviation, BMI = body mass index; CIS = carcinoma in situ; ECOG = Eastern Cooperative Oncology Group

**Fig 1:**
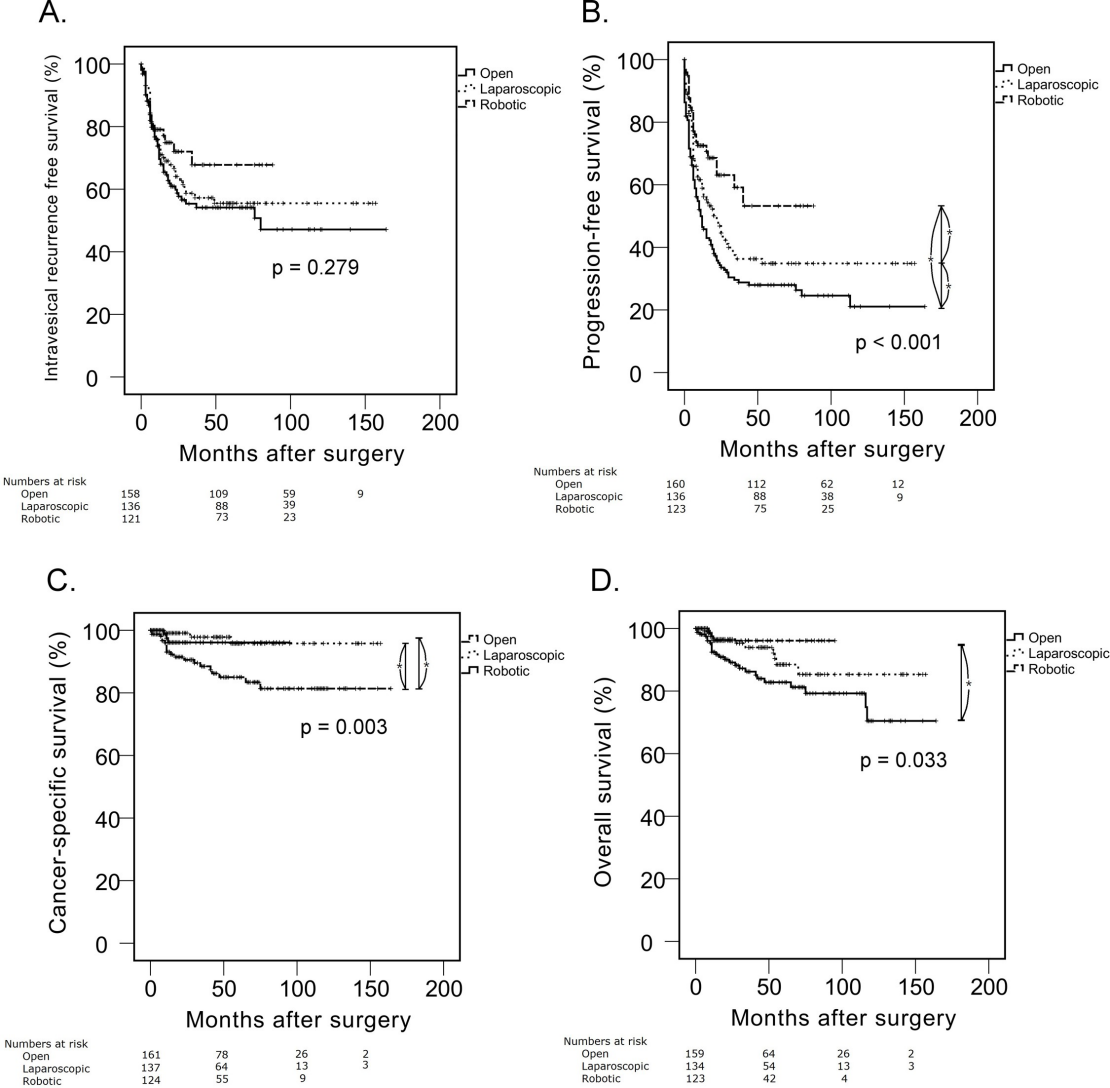
Kaplan-Meier analysis of survival outcomes of patients treated with nephroureterectomy for non-metastatic upper tract urothelial cell carcinoma.

These results were associated with significantly shorter postoperative survival in the open approach than in the other approaches in progression-free (p < 0.001), cancer-specific (p = 0.003), and overall survival (p = 0.033) (Fig 1). Interestingly, there was no significant difference in postoperative intravesical recurrence among the three groups (p = 0.279). Subsequent multivariate Cox proportional analyses showed no significant associations between surgery type and postoperative survival (all p-values > 0.05) (Table 3). When we analyzed the postoperative survival outcomes only in patients with longer follow-ups (≥ 24 months), the type of surgery did not show any significant associations with oncological outcomes (all p-values > 0.05).

**Table 3:** Multivariate Cox proportional hazards analyses for surgical approaches estimating each survival outcome.

		HR (95% CI of HR)	p value
		N = 422	
	Open	Reference	
Intravesical recurrence	Laparoscopic	0.803 (0.543–1.186)	0.270
	Robotic	0.665 (0.405–1.092)	0.107
Progression	Laparoscopic	0.850 (0.627–1.152)	0.296
	Robotic	0.574 (0.276–1.325)	0.326
Overall mortality	Laparoscopic	0.537 (0.248–1.163)	0.115
	Robotic	0.335 (0.097–1.158)	0.084
Cancer specific mortality	Laparoscopic	0.364 (0.104–1.282)	0.116
	Robotic	0.336 (0.070–1.607)	0.172

All multivariate analyses were adjusted by following variables: age, sex, body mass index, ECOG score, T stage, Tumor size, cellular grade, Charson cormorbidity index, multifocality of tumor, lymphadenectomy and lymph node invasion

Furthermore, we tried to compare the survival outcomes according to the different salvage chemotherapies (gemcitabine/cisplatin, methotrexate/vinblastine/doxorubicin/cisplatin, paclitaxel, combination). However, there was no significant difference between the subgroups [overall survival: p = 0.837, cancer-specific survival: p = 0.789].

When we compared the perioperative outcomes among the groups, we found no significant differences in intraoperative and postoperative complication rates (all p-values > 0.05) (Table 4). Furthermore, when we compared the postoperative complications according to the Clavien-Dindo classification, no significant differences were observed among the groups (p = 0.664) (Table 5). Conversely, patients who were treated by the laparoscopic or robotic approach experienced significantly less blood loss during surgery (p = 0.001) and had a shorter hospital stay (p < 0.001) than patients treated with the open approach (Table 4). Moreover, the laparoscopic and robotic groups required significantly less analgesic usage than the open approach despite absence of significant differences in postoperative pain scores (p > 0.05).

**Table 4:** Peri-operative outcomes according to type of surgery.

	Open surgery	Laparoscopic surgery	Robotic surgery	p value
Operation time (min)	210.5 ± 72.2	230.2 ± 77.9	248.5 ± 79.3	< 0001
Intraoperative complications	13 (8.1%)	7 (5.1%)	9 (7.3%)	0.363
Postoperative complications	24 (14.9%)	20 (14.6%)	17 (13.7%)	0.880
Estimated blood loss (during surgery)(ml)	339.4 ± 346.8	261.1 ± 330.8	200.5 ± 198.3	0.001
Length of hospital stay (days)	12.8 ± 5.0	10.4 ± 3.7	10.3 ± 4.9	< 0.001
Amount of postoperative analgesics usage (times)	2.1 ± 3.1	1.5 ± 2.0	1.5 ± 2.2	0.015
Postoperative pain score: POD #1	4.4 ± 1.6	4.6 ± 1.4	4.4 ± 1.1	0.360
Postoperative pain score: POD #7	1.1 ± 1.4	1.1 ± 1.4	1.2 ± 1.2	0.382

**Table 5:** Differences of post-operative complications according to type of surgery in the patients with upper tract urothelial cancer.

Clavien-Dindo classification	Open surgery	Laparoscopic surgery	Robotic surgery	p value
No complication	137 (81.1%)	117 (85.4%)	107 (86.3%)	0.664
Grade I	10 (6.2%)	3 (2.2%)	5 (4.0%)	
Grade II	12 (7.5%)	15 (10.9%)	11 (8.9%)	
Grade III	2 (1.2%)	2(1.5%)	1 (0.8%)	
Grade IV or higher	0 (0%)	0 (0%)	0 (0%)	

## Discussion

In this study, we observed similar complication rates among the three surgical approaches (open/laparoscopic/robotic) intraoperatively and postoperatively. However, the laparoscopic and robotic approaches showed better perioperative outcomes, such as significantly less bleeding and a shorter hospital stay, than the open approach. Although we could not find any difference in postoperative pain using the visual analog score for pain, patients treated with laparoscopic and robotic approaches required significantly less postoperative analgesics during the postoperative recovery period. However, the type of surgical approach was not significantly associated with postoperative survival outcomes.

During the past couple of decades, laparoscopic surgery has become a standard treatment option for various benign and malignant urologic diseases [12]. Laparoscopic surgery has confirmed technical feasibility and better perioperative outcomes (less postoperative pain, rapid convalescence, shorter hospital stay) compared with the open approach [13]. However, robotic surgery has also been used for various urological surgeries such as radical prostatectomy, nephrectomy, and pyeloplasty [14]. Several advantages of the robotic system have overcome several obstacles of conventional laparoscopic surgery. The increased dexterity and accuracy, improved visualization, and more ergonomic position of robotic surgery have made it a major surgical approach in contemporary urology. These minimally invasive techniques have also been adopted for nephroureterectomy [15–17]. Although laparoscopy was sufficiently compared with open surgery in previous studies, robotic nephroureterectomy was not. Furthermore, the three surgical approaches were not simultaneously compared in the same clinical setting. In the present study, we tried to evaluate the various clinical outcomes of the three surgical approaches in patients who underwent nephroureterectomy to treat localized UTUC.

Previous studies also showed several advantages of minimally invasive surgeries in patients who underwent nephroureterectomy [18–20]. In 2004, Rassweiler et al. performed a systematic review of 85 studies including 1,365 patients [18]. They found that the laparoscopic group had a slightly longer operation time (276.6 versus 220.1 min) and significantly lower blood loss (240.9 versus 462.9 mL) than the open group. The progression-free 2-year survival rates were similar (75.2% versus 76.2%) between groups. They concluded that laparoscopic nephroureterectomy can provide some advantages of minimal invasive surgery with similar oncological control. Another study by Capitanio et al. compared open and laparoscopic nephroureterectomy in 1,249 patients with non-metastatic UTUC [19]. Surgery type was not associated with recurrence-free or cancer-specific survival in their multivariate analysis. Although their study evaluated only short-term oncological data between laparoscopic and open nephroureterectomy, the two groups showed similar oncological outcomes.

Robotic nephroureterectomy is relatively under-studied compared with open and laparoscopic approaches. Ambani et al. analyzed the result of 27 robotic and 20 laparoscopic nephroureterectomies and presented the initial experiences of the robotic approach [20]. They found that the robotic approach was associated with longer operation time and much greater blood loss (all p-values < 0.05) than the laparoscopic approach. Subsequently, Melquist et al. compared 37 patients treated with robotic approaches to 63 patients treated with laparoscopic approaches and concluded that the robotic approach was associated with improved lymph node procurement and a lower risk of major bleeding [21]. However, robotic surgery showed significantly longer operation time (5.1 versus 3.9 h, p = 0.001) and longer hospital stay (5.0 versus 4.0 days, p = 0.002). In our study, of the three approaches, robotic surgery had the longest operation time. We believe that this is because of the longer preparation times during the initial docking process of the robot and we sometimes needed to re-dock the robotic system according to the type of bladder cuffing, which also led to further delay. However, when we compared operation times between the each approach using independent t test, there was no significant differences between the laparoscopic and robotic approaches (p = 0.061). Furthermore, when we divide the robotic group into three subgroups in chronological order, there were significant differences between the first group and the other two groups (first group, 293 min; second group, 225 min; third group, 233 min, p < 0.001). We believe that the operation times were reduced according to the accumulation of experiences after the first 42 cases. Similar findings were also observed for other perioperative outcomes (blood loss, length of hospital stay, postoperative analgesic usage). Although open surgery showed significantly inferior outcomes compared with the other two approaches in those three variables, the laparoscopic and robotic approaches did not differ significantly (all p-values > 0.05). These findings imply that the laparoscopic and robotic approaches share the advantages of minimally invasive surgery.

In our study, robotic nephroureterectomy showed significantly longer progression-free, cancer-specific, and overall survival (all p-values < 0.05) compared to open surgery on univariate Kaplan-Meier analysis. However, we believe that those results do not indicate superior oncological outcomes of the robotic approach because patients treated with it had significantly favorable clinical characteristics including tumor size, multifocality, and stage. Furthermore, multivariate Cox analysis showed no significant associations between surgery type and survival outcomes (all p-values > 0.05). A similar finding was observed in a previous study by Kido et al. who compared the laparoscopic and open approaches [22]. In their study, the open approach showed significantly shorter progression, cancer-specific, and overall survival on univariate Kaplan-Meier analysis. However, subsequent multivariate Cox regression tests showed no significant differences in survival outcomes. We believe that the robotic and laparoscopic approaches can provide at least equivalent oncological outcomes to those of the open approach.

Despite our result, there are still concerns about the cost-effectiveness of robotic surgery compared with open/laparoscopic surgery. Yun et al. recently compared the overall cost between robotic/laparoscopic/open prostatectomy in patients with localized prostate cancer in South Korea. Briefly, they reported that the cost of robotic surgery was approximately two-fold to three-fold higher than that of laparoscopic and open prostatectomy [23]. The median price for robotic prostatectomy was 14,253 US dollars (laparoscopic prostatectomy: 4,073 US dollars, open prostatectomy: 1,599 US dollars) in their study. Another study by Chang et al. retrospectively analyzed data from 489,369 subjects in the United States between 2003 and 2010 [24]. In 2003, the median costs for robotic and open surgery were 16,358 and 7,814 US dollars, respectively. Interestingly, the cost of robotic surgery decreased to 10,622 US dollars in 2010, but the cost of open surgery increased to 9,832 US dollars. Accordingly, the difference in costs between robotic and open surgery continuously decreased annually in their study. We believe that the cost for robotic surgery in South Korea is still high compared to that in the United States, but further competition in the market of a robotic surgical system will hopefully reduce the cost of robotic surgery in the near future.

The present study has certain limitations. First, as a retrospective study, it has inherent selection bias. For example, patients undergoing the open approach showed significantly worse pathologic profiles than did those on laparoscopic and robotic approaches. Therefore, our results should be validated by further prospective randomized controlled study for better comparison of oncological outcomes of minimal invasive therapies. Second, the method of bladder cuff excision was not unified according to each approach, introducing a possible confounding effect. Third, our follow-up periods were not sufficient to evaluate the long-term survival outcomes, and the robotic group had significantly shorter follow-up periods than did the other groups. Moreover, different salvage treatments among the patients might influence the survival outcomes in the present study. Even though our subgroup analysis based on chemotherapy regimen did not show any between-group survival differences, the number of our study subjects was too small and our results may be seriously biased. Therefore, further prospective randomized studies with a larger cohort are needed to validate our results.

## Conclusions

Minimally invasive surgeries (laparoscopic/robotic) showed some better perioperative outcomes after radical nephroureterectomy in patients with non-metastatic UTUC. However, the oncological outcomes should be validated by further prospective studies with longer follow-up periods.
